# Single Atom Catalyst for Nitrate‐to‐Ammonia Electrochemistry

**DOI:** 10.1002/smll.202403515

**Published:** 2024-09-30

**Authors:** Suvani Subhadarshini, Martin Pumera

**Affiliations:** ^1^ Future Energy and Innovation Laboratory Central European Institute of Technology Brno University of Technology Purkyova 123 Brno 61200 Czech Republic; ^2^ Faculty of Electrical Engineering and Computer Science VSB – Technical University of Ostrava 17. listopadu 2172/15 Ostrava 70800 Czech Republic

**Keywords:** ammonia, catalysis, electrochemistry, single atom catalysis

## Abstract

Various life forms suffer from the negative effects of nitrate when it accumulates in water bodies, which is a major concern in the present day. The removal of nitrate from water bodies is a critical challenge, and the most effective method to achieve that is to change it into ammonia. Ammonia is a clean energy source and a vital input for the fertilizer industry. The Haber–Bosch process, which dominates the industrial production of ammonia, requires a lot of energy. A more sustainable way to produce ammonia is to use nitrate‐contaminated water and reduce it to ammonia through electrocatalysis. This review is constituted of amalgamated articles featuring unique conditions that affect the productivity and activity of the transition metal single atom catalyst (TNMSAC) for the electrocatalytic nitrate reduction to ammonia (NRA) reaction. It explores factors such as nitrate ion adsorption, the characteristics of the central electroactive transition metal, the type of coordinating atoms, the impact of potential on stability, and the interplay among single atoms on the selectivity and yield of ammonia gas. In addition, this review also covers advanced concepts such as dual‐atom catalysts, dual single atom catalysts, and single atom alloys. The review will provide valuable guidance for enhanced comprehension and strategic designing of TNMSAC for the electrocatalytic conversion of NRA, which will contribute to achieving a green ammonia economy.

## Graphical abstract

This review constitutes articles which feature unique conditions that affect the productivity and activity of the transition metal single atom catalyst for the electrocatalytic nitrate to ammonia reaction. Factors such as nitrate ion adsorption, characteristics of the central electroactive transition metal, type of coordinating atoms, impact of potential on stability, and the interplay among single atoms on the selectivity and yield of ammonia have been explored.

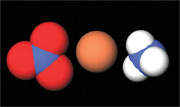

## 1 Introduction

Industrial human activities have disturbed the balance in the surrounding ecosystems, affecting the lives of living organisms. One of the vital natural cycles that is disrupted is the one involving nitrogen.^[^ ^1^, ^2^, ^3^ ^]^ The unreasonable release of water from agricultural fields, sewage leachate, domestic waste water, and industrial wastes rich in nitrogen oxides (especially nitrates) wreaks havoc when mixed with water bodies, resulting in artificial eutrophication. The eutrophication then triggers algal blooms, which lead to the death of aquatic life forms due to lack of oxygen and sunlight. When such polluted water is used for drinking by higher organisms like humans, it causes diseases that worsen health conditions in the long term.^[^ ^4^, ^5^ ^]^ The situation is so serious that the United States Environmental Protection Agency and the European Parliament have declared nitrate contamination as a matter of utmost priority, and have set essential goals to eliminate it.

To address the worsening unbalance, the scientific community has been working to retrieve nitrate ions from water bodies and progress with its accurate sequestration. Various solutions have been offered to combat the issue, e.g., transition metal oxides,^[^ ^6^, ^7^, ^8^, ^9^, ^10^, ^11^, ^12^ ^]^ transition metal dichalcogenides,^[^ ^13^, ^14^, ^15^, ^16^, ^17^, ^18^, ^19^, ^20^ ^]^ carbon‐based materials,^[^ ^21^, ^22^, ^23^, ^24^ ^]^ C_3_N_4_,^[^ ^25^, ^26^, ^27^, ^28^, ^29^, ^30^ ^]^ and composites^[^ ^31^, ^32^, ^33^, ^34^, ^35^, ^36^, ^37^ ^]^ have been extensively studied for their electrocatalytic behavior toward nitrate reduction to ammonia (NRA). This kind of electro‐reduction accentuates the notion of developing treasure from trash. Ammonia is a value‐added product that promotes the vision of carbon‐neutral energy and sustainable development. It is the source of the carrier gas for H_2_ as well as N_2_. Ammonia is mainly produced using the Haber–Bosch Process (HBP). The HBP stands as the industrial benchmark for ammonia production but is plagued by the high energy requirements to generate the high temperature and pressure conditions needed for the catalytic process. The Haber–Bosch process is responsible for about 2% of worldwide energy consumption.^[^ ^38^ ^]^ The overall process is not sustainable due to its high energy demand to maintain the extreme operational conditions. An alternative to the HBP is the electrochemical production of ammonia at room temperature, which does not require maintaining high temperature and pressure conditions. Hence, it can be seen as a sustainable substitute for the HBP for ammonia production.^[^ ^39^ ^]^

Electrochemical NRA involves a series of electron and proton transfer reactions. The transfers take place in a step‐wise manner. However, the main challenge in the general solutions proposed by the NRA electrocatalysts is the absence of a clear mechanism of interaction between the catalyst and the nitrate ions. The multi‐step reaction produces some undesired intermediates, which may reduce the efficiency of ammonia production and affect its yield significantly.^[^ ^40^, ^41^, ^42^, ^43^, ^44^ ^]^ Catalysts without a specific interaction mechanism may produce unwanted side products by reacting with intermediates.^[^ ^45^ ^]^ To address the problem of a regulated or guided mechanism, the research focus shifted to the use of single atom catalysts (SACs) for NRA. An SAC, as the name implies, is a catalyst composed of single metal atoms. Typically, single atom is coordinated with chelating atoms, which secure it and attach it to the matrix where it is dispersed. They are supposed to increase efficiency due to the controlled interaction of nitrate ions and low chances of unwanted by‐product formation. Another astonishing fact about SACs is that their catalytic property can be altered by the controlled change of the heteroatoms that anchor them to the matrices.^[^ ^46^, ^47^, ^48^, ^49^, ^50^, ^51^ ^]^ SACs for NRA are expanding at an appreciable rate as they have attracted the attention of many researchers due to their high catalytic efficiency.^[^ ^52^ ^]^ Considering the intriguing attention directed toward this novel field, we have collected a handful of research works that are unique in terms of strategically directing catalytic conditions to improve the adsorption, selectivity, and yield in the NRA reaction. Directing the catalytic parameters involves considering the coordination symmetry of the catalyst, alterations with the applied potential, experimenting with the central atom, chelating groups, and support matrix, the formation of a single metal alloy, use of diatoms, and the application of dual single atoms.

## 2 Nitrate to Ammonia Conversion

### 2.1 Theoretical Studies of Nitrate to Ammonia Conversion by Transition Metal Single Atom Catalyst

The choice of transition metal single atom catalyst (TNMSAC) for NRA is rather vast. Several recent papers have employed computational methods to identify the optimal transition metal embedded in different matrices, such as graphene sheets, carbon–nitrogen frameworks, or N/P/S‐doped conjugated organic frameworks. Wang et al.^[^ ^53^ ^]^ recently performed a comprehensive analysis of the catalytic performance of various TNMSACs using a high‐throughput screening method. They applied a four‐step screening process to select or eliminate the best TNMSAC on a nitrogen‐doped graphene sheet (NDG). A series of transition metals were computationally assessed for their nitrate reduction performance. The selection/elimination process was based on the following factors: (i) the stability of the TNMSAC was thoroughly investigated to ensure that the synthesized SAC could withstand the experimental conditions and remain stable throughout the experiment, (ii) the adsorption capacity of the catalyst surface for the nitrate species in the solution was examined. The catalyst performance largely depended on how effectively the nitrate ions were attached to the electrocatalytically active surface of the catalyst, and (iii) the reactivity of the catalyst, which was the most important performance factor, was evaluated. Finally, the TNMSACs were also compared based on the amount of ammonia they produced, without generating any unwanted by‐products like nitrite (selectivity). Thus, the entire screening process was based on the four key aspects of catalytic strength, stability, selectivity, and performance.

In this work, the authors attempted to create a stable framework by introducing a transition metal into the graphene framework, coordinated with four nitrogen atoms that replaced two carbon vacancies. The sizes of the transition metals were kept in consideration while examining their stability in the NDG framework. The binding energy of the various transition metals was calculated to further verify their stability. The binding energy of the TNMSAC on NDG was the difference between the energy of the TNMSAC and the sum of the energies of the four coordinated nitrogens and the transition metal. The transition metals were chosen based on the stability of the SAC catalyst derived from the binding energy values. Negative binding energy values indicated stable TNMSAC catalysts. Ag‐SAC showed a positive binding energy value in the screening process, implying its instability issues. The adsorption efficiency of nitrate ions on the surface of transition metals was evaluated as a criterion for selecting the best catalyst. The underlying principle assumed that the lower the free energy of the adsorption process, the higher the likelihood of the nitrate ions undergoing reduction to produce ammonia. Therefore, the nitrate ions could adsorb on the surface of the TNMSAC while supported by the NDG framework in two distinct modes: one involving the coordination of a single oxygen atom and the other involving the coordination of both oxygen atoms. The thermodynamic feasibility of the process depended on the free energy of the adsorption mode. The mode involving the coordination of both oxygen atoms had lower free energy than the mode involving a single oxygen atom. Another important parameter was that the free energy of nitrate adsorption on the TNMSAC should be lower than that of hydrogen or nitrogen adsorption. A series of transition metals was screened and it was observed that most of the first‐row transition metals had lower free energy values for nitrate adsorption than for hydrogen or nitrogen adsorption.

The mode of interaction of nitrate with the catalyst for its efficient reduction to ammonia gas was postulated based on the simulation (high‐throughput screening) calculations. The study suggested that the initial step was the adsorption of NO_3_^−^ ions on the surface of the TNMSAC. The free energy change values of individual components determined the direction of the reaction pathways, *i.e*., the lower the free energy, the more favorable the reaction pathway. The calculation revealed that some TNMSACs (Fe and Os) had optimal values of Gibbs free energy, making them suitable candidates for nitrate reduction to ammonia. The values indicated that they did not facilitate the strong binding of nitrate to the electroactive surface or the other way around. Therefore, from the high‐throughput screening assessment, the candidates were selected as the best among the remaining 23 different transition metals under study.

The optimal value of ∆G for nitrate adsorption is moderate for electrochemical reduction studies. A reaction with a very low ∆G value will cause strong adsorption, which will impede the reaction's progress. Conversely, a reaction with a very high ∆G value will cause weak adsorption, which will reduce ammonia production. Finally, the ease of releasing the formed ammonia from the active centers of the fabricated TNMSACs was assessed based on the positive values of ∆G. Therefore, a higher value of ∆G indicates better desorption of the produced ammonia gas. The electrocatalytic production of ammonia by both Fe‐ and Os‐SACs is facilitated by their positive ∆G values. To evaluate their selectivity for ammonia gas over by‐products, the two TNMSACs were compared. The results showed that Os‐SAC had a higher selectivity than Fe‐SAC because the ∆G values of hydrogen evolution were much lower than those of ammonia formation for the latter. The high‐throughput screening evaluation revealed that Os‐SAC was the most effective among the 23 transition metal single atom catalysts examined for electrocatalytic ammonia production from nitrate ions. This can be explained by the fast charge transfer from the d orbitals of the Os and Fe single atoms, which alters the nitrogen–oxygen bond length. The former has a greater charge transfer, resulting in higher ammonia yield and selectivity.

**Figure 1:**
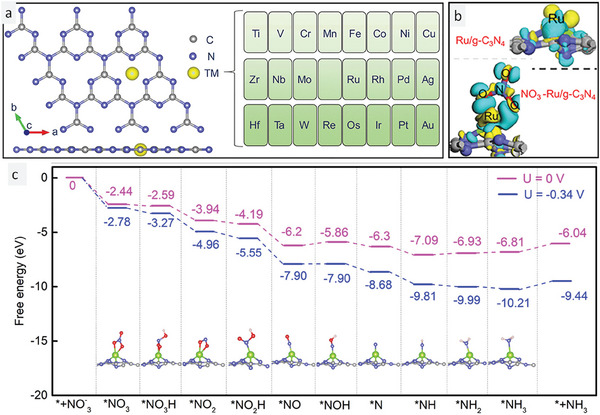
Theoretical approaches for the determination of the ideal SAC atom chelation on g‐C_3_N_4_ and the reaction mechanism. a) TNMSAC anchored on graphitic carbon nitride (GCN) structure. b) Electron density distribution across Ru‐g‐C_3_N_4_ without and with the adsorption of nitrate ions. c) Mechanism of conversion of nitrate to ammonia by Ru‐g‐C_3_N_4_. Reprinted with permission from ref. [54]. Copyright 2021 American Chemical Society.

Using a similar simulation approach, Lv et al.^[^ ^54^, ^55^ ^]^ evaluated the performance and selectivity of six different TNMSACs supported on graphitic carbon nitride (GCN) for the electrocatalytic conversion of nitrate to ammonia (**Figure** **1**(a)). They applied the first principle calculation (FPC) method to select the best TNMSAC catalyst from various TNMs such as titanium, osmium, ruthenium, chromium, manganese, and platinum. They chose GCN as the backbone/support material for the TNMSACs due to its inherent cavity that could host and stabilize the single atoms, moreover, the nitrogen coordination in the GCN framework facilitated the proper chelation and enhanced the coordination stability of the single atoms within the GCN network. The authors hypothesized that GCN was a superior stabilizing matrix to pure graphene sheets owing to its structural and chemical features. They also performed a detailed study on the adsorption of nitrate ions on the TNMSAC/GCN surface. The FPC‐derived rate‐determining steps helped elucidate the reaction mechanism. The feasibility of the reaction depended on two key parameters: the potential of the rate‐determining step and the overall ∆G of the reaction steps. The reaction was more feasible when the limiting potential of the electrochemical reaction was higher and the ∆G was lower (negative).

The stability and performance of the TNMSACs under investigation were evaluated based on their energy of formation and potential for dissolution. The FPC studies revealed that the more stable TNMSACs had a negative–positive combination of these values. The ∆G was another critical parameter. Some TNMSACs, such as iron and cobalt, had a positive ∆G, which implied thermodynamic infeasibility. Therefore, this method was effective in eliminating the unstable transition metals that would not undergo a favorable transition in the electrocatalytic reduction process.

The pristine GCN framework was used as a substrate and FPC was applied to examine nitrate ion adsorption on its surface. The results showed a positive and large ∆G value, indicating the non‐spontaneity of the chosen process. Therefore, the system only became active for nitrate adsorption and subsequent electrocatalytic ammonia production when the GCN matrix was modified with TNMSAC. The charge redistribution on the TNMSAC/GCN surface upon nitrate ion adsorption was also studied in detail. The electron density was transferred from the GCN substrate to the transition metal SAC center and then to the adsorbed nitrate ion. This study showed that the GCN provided the electron density that moved through the SAC and activated the nitrate ion, enabling the electrocatalytic production of ammonia. A control simulation study was also performed to examine the charge distribution in a system with TNMSAC on GCN without nitrate ions. The FPC indicated that the electron density shifted from the TNMSAC center to the GCN substrate. This study demonstrated the cooperative effect of GCN, TNMSAC, and nitrate ions in the joint transfer and rearrangement of electron density across the system, which facilitated the electrocatalytic conversion of nitrate to ammonia gas. The charge movement in the system is marked with two different colors for better clarity. The blue and yellow colors indicate the regions of low and high density, respectively (Figure 1). To identify the best TNMSAC catalyst among the six TNMSACs examined, the authors performed simulations to calculate the partial density of states (PLDYS) after establishing the significance of the TNMSAC/GCN system for effective electrocatalytic nitrate reduction. The PLDYS revealed the electronic structure of the reaction components and showed that pristine GCN was a semiconductor while TNMSAC on GCN was a metal, indicating a transition to a more conductive system that is favorable for electrocatalytic nitrate‐to‐ammonia conversion. To study the adsorption affinity of nitrate ions on the TNMSAC surface, the d‐band center theory was applied. The theory showed that ruthenium had the highest positive value among the six transition metals. Therefore, the PLDYS and the d band theory confirmed that the ruthenium TNMSAC on GCN had the best electrocatalytic performance for nitrate reduction to ammonia. The authors suggested a mechanism with two rate‐determining steps (RDS) to measure the catalytic activity of each TNMSAC. The first RDS was the formation of activated NOH by the hydrogenation of activated NO with a proton and electron pair, the second RDS was the production of NH_3_ by the reduction of activated NH_2_ with a proton and an electron. The rate‐limiting potential values for titanium, osmium, ruthenium, chromium, manganese, and platinum TNMSAC on GCN were −0.78, −0.72, −0.34, −0.67, −0.86, and −0.91 V, respectively. Ru‐SAC had the highest limiting potential and thus needed the least extra potential for the electrochemical conversion of nitrate to ammonia. Ru‐SAC also had a moderate nitrate ion adsorption that did not interfere with the reaction by blocking the 1^st^ rate determining step (RDS) (weak adsorption) or the 2^nd^ RDS (strong adsorption).

The two articles mentioned above offer various insights. First, despite both using simulation studies to calculate and screen the catalysts, they each proposed different mechanisms of action. These mechanisms are distinct and have different RDSs that govern the nitrate‐to‐ammonia conversion. Second, the substrates (NDG and GCN) for both articles are similar (graphene‐based frameworks), but the ∆G_(adsorption)_ values for nitrate ion adsorption on the surface vary greatly. Third, the central metal of the single atom catalyst is not the sole factor that determines its activity in electrocatalytic nitrate reduction. For example, in the NDG system, osmium SAC performed the best while in the GCN framework, ruthenium SAC was the most effective for nitrate reduction. This observation suggests that the performance of the catalyst depends not only on the central group but also on its interaction with the surrounding coordination groups and its electronic structure.

It was recently found^[^ ^55^ ^]^ that TNMSAC systems supported on nitrogen‐doped graphitic sheets showed doping‐dependent catalytic activity for the electrochemical conversion of nitrate to ammonia. The authors utilized a theoretical‐based approach for the screening of transition metals belonging to the 3d, 4d, and 5d series, and found that the simulation studies for the initial step—the adsorption of nitrate ions—was at the optimum when the graphitic sheets had multiple defects. The defects triggered the efficient adsorption of the nitrate ions onto the catalyst surface. Upon simulating the energy of adsorption, it was found that the TNMSAC centers with single nitrogen‐doped centers showed higher energy of adsorption, indicating a lower efficiency as a catalytic material. Instead, the TNMSAC centers with two pyridinic nitrogen showed better catalytic adsorption results. The best adsorption activity was, however, shown by TNMSAC with two nitrogen‐doped graphitic carbon frameworks with the orientation of both nitrogen's *trans* to each other. Hence, this work throws light on the fact that TNMSACs can be screened for their NTA efficiency not only based on the central atom but also by the number of nitrogen atoms and their positions on the graphitic carbon framework that holds the transition metals. Thus, two pyridinic nitrogen‐doped graphitic matrices were established as the best catalyst matrix for holding the SAC for the NTA reaction. In another work, Liu et al.^[^ ^56^ ^]^ showed that the introduction of nitrogen within the coordination environment of the catalytic system helps enhance NRA activity of the TNMSAC. They proved their hypothesis on the addition of nitrogen to the backbone matrix of the Cu‐TNMSAC through experimental data. To prove their point, a control system was chosen without the incorporation of nitrogen. This system, consisting of pyridinic N‐coordinated Cu‐TNMSAC, showed lower ammonia yield, faradaic efficiency (FE) of 95%, and high yield of 131 mg_NH3_ mg^−1^ h^−1^.

### 2.2 Experimental Studies of Nitrate to Ammonia Conversion by Transition Metal Single Atom Catalyst

A specific type of TNMSAC has been the subject of many recent works that synthesize it and test its efficacy in reducing nitrate to ammonia electrochemically. These experimental works differ from simulation studies that examine a range of TNMSACs as they provide detailed evidence for the catalytic performance of only one or two. Rather than reviewing the usual publications on the electrochemical production of ammonia, we have selected a seminal works that stand out for their attention to the fine details of the experiment and the reaction mechanism of the nitrate reduction to ammonia. The synthesis of ammonia by iron TNMSAC (Fe‐TNMSAC) was the main focus of Wu et al.^[^ ^57^ ^]^ The authors addressed the problem of non‐selective product formation by conventional catalysts. The complex mechanism of nitrate reduction to ammonia leads to various unwanted by‐products that reduce efficiency significantly and make the catalysis approach unsustainable due to the high energy loss.

Iron was the chosen catalyst because of its high efficiency in nitrogen‐fixing enzymes (bacteria) in the soil that sequester nitrate contaminants and transform them into valuable ammonia. Haber and Bosch devised the industrial production of ammonia using iron as a catalyst.^[^ ^58^, ^59^, ^60^ ^]^ Therefore, the authors selected iron from all the transition metals to synthesize Fe‐TNMSAC as it has a proven track record of producing ammonia. The authors also noted that conventional catalysts make it difficult to unravel the mechanism and selectivity of the product formation. The difficulty stems from a poor understanding of the multiple elementary steps involved in the mechanism. Thus, the authors suggested that single iron atoms could facilitate the comprehension of the elementary steps that produce ammonia.

**Figure 2:**
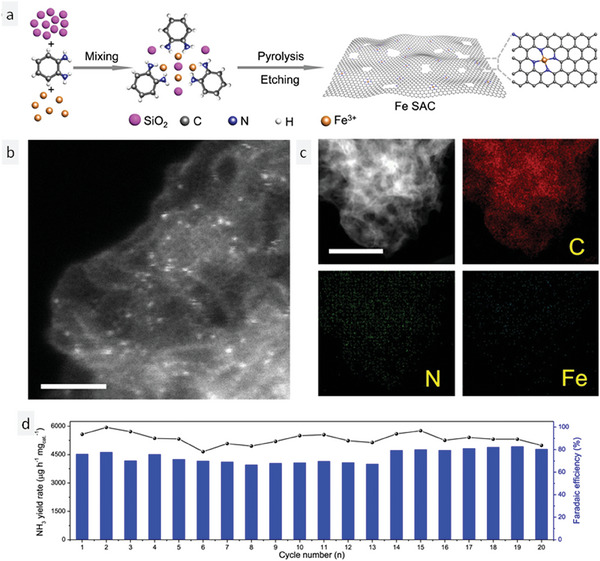
Application of Fe‐TNMSAC for the electrochemical reduction of nitrate to ammonia: synthesis, characterization, and catalytic stability. a) Schematic representation of the synthesis process of Fe‐TNMSAC. b) HAADF‐STEM micrograph of Fe‐TNMSAC. c) EDX analysis showing the elemental distribution of iron, nitrogen, and carbon in Fe‐TNMSAC. d) Cycling stability of Fe‐TNMSAC. Reprinted with permission from ref. [57]. Copyright 2021, Springer Nature.

Using silicon dioxide hard templates, Fe‐TNMSAC was synthesized by nucleating and growing the seeds. Ferric chloride and ortho‐phenylenediamine were the precursors for iron and the carbon–nitrogen support matrix. The precursors were uniformly mixed with silicon dioxide in isopropyl alcohol solvent and magnetically stirred for 12 h. The mixture was dried in a rotary evaporator and then pyrolyzed at 800 °C for 2 h under argon gas. The mixture was then freed from silicon dioxide by alternating baths of 2 M NaOH and 2 M H_2_SO_4_. Finally, the etched mixture was pyrolyzed again as before to obtain the Fe‐TNMSAC. **Figure** **2**a is a schematic representation of the synthesis process. The transmission electron microscope (TEM) micrographs in Figure 2b characterize the as‐synthesized Fe‐TNMSAC and reveal the iron SAC distribution on the carbon matrix. The energy dispersive X‐ray (EDX) analysis in Figure 2c displays the elemental composition of iron, nitrogen, and carbon in the sample. The electrocatalytic reduction of nitrate to ammonia was initially assessed using linear sweep voltametry (LSV) curves with two different electrolyte solutions—potassium sulphate (PS) and PS+ potassium nitrate (PN) using glassy carbon with Fe‐TNMSAC as the working electrode. The LSV curves exhibited a significant drop in the current density value, implying the reduction of nitrate ions in the electrolyte. The LSV curve for the blank electrolyte had a higher current density, indicating no reduction reaction. To measure the amount of ammonia produced, long‐term electrolysis experiments were performed for 30 minutes over a potential range of −0.50 V versus RHE to −0.85 V versus RHE.

The authors further demonstrated the importance and excellence of Fe‐TNMSAC by comparing it with other Fe‐based nano systems. The ammonia yield rates of Fe‐TNMSAC, Fe nanocluster, and Fe nanoparticle‐nanocluster were compared. As expected, Fe‐TNMSAC had the highest ammonia yield followed by Fe nanoparticle‐nanocluster and Fe nanocluster. The authors also quantified the electrocatalytic nitrate reduction performance of different transition metals relative to Fe‐TNMSAC. The ammonia yield rate of two other transition metal SACs, Co‐TNMSAC, and Ni‐TNMSAC, was experimentally confirmed. The ammonia yield of the transition metals was in the order of Fe, Co, and Ni. Fe‐TNMSAC achieved an ammonia yield rate of 1.0 mol s^−1^ mol_m_^−1^ at a potential of −0.8 V versus RHE. The authors also performed a cyclic stability test for Fe‐TNMSAC by repeating the electrolysis experiments on the same catalyst for 20 cycles. The cyclic stability test results were impressive as 80% FE was attained after 20 cycles (Figure 2d).

To prove one of the most important hypotheses proposed by the authors regarding the selectivity of the Fe‐TNMSAC, density functional theory calculations were used to screen off the elementary reactions with positive ΔG. First, all the elementary reactions leading to the formation of ammonia and other by‐products such as nitrogen, nitric oxide, and nitrous oxide were studied. Then, step‐by‐step, the ΔG of the individual steps were calculated and the feasibility was tested. The reduction initiates with the protonation of the adsorbed nitrate ion on the surface of the catalyst. The solvent (electrolyte) acts as a proton source. The second step proceeds with the attack of a pair of protons and electrons on the surface of the catalyst to yield activated NO_2_ species. The third step also proceeds like the second step with the attack of a pair of protons and electrons on activated NO_2_ to form HNO_2_ in the activated state. The fourth step proceeds with the addition of a proton and electron on activated HNO_2_ to form NO, which enables further attack by another proton–electron pair, yields activated HNO species. The fifth step proceeds with the formation of activated N species on the surface of the catalyst. This step is highly crucial as it deals with the specificity of the catalyst. The essence of the present work revolves around the concept of high specificity of the catalyst (Fe‐TNMSAC) as proposed by the authors. Thus, at this point, the authors propose that there stands a possibility that the activated N species on the surface of the Fe‐TNMSAC (N*@Fe‐TNMSAC) has a very high probability of combining with another activated N species on the catalyst surface to yield nitrogen gas. From a thermodynamic perspective, we know that the entropy of a gas is positive and its randomness is high. For this reason, a reaction becomes highly spontaneous if the products it yields are in a gaseous state. Hence, the combination of two units of N*@Fe‐TNMSAC to form nitrogen gas can be seen as a highly competitive process in comparison to the usual protonation process that would yield NH activated species. However, there is an essential prerequisite condition that must be satisfied for the two units of N*@Fe‐TNMSAC to interact with each other. The essential condition is that for the two activated N to interact with each other, they should lie near each other. Only when the condition of proximity is satisfied will the interaction yield nitrogen gas. However, the authors stated that they designed the Fe‐TNMSAC in such a way that the two units of N*@Fe‐TNMSAC can never be close to each other. The authors justified their strategy by stating that the distribution of iron single atoms on the surface of the carbon–nitrogen framework is sparse and they are arranged on the matrix in such a fashion that they have a large distance between the two Fe single atoms. Hence, they proceed to justify the hypothesis that the production of nitrogen gas by‐product has been prevented. Thus, it can be shown that the catalysts are highly selective in yielding ammonia. The next step involves the protonation of the activated N to form NH‐activated species. The last two steps directly involve two successive protonations of the activated NH species to yield NH_3_ a major product.

### 2.3 The Effect of Coordination Symmetry of Transition Metal Single Atom on NRA Activity

**Figure 3:**
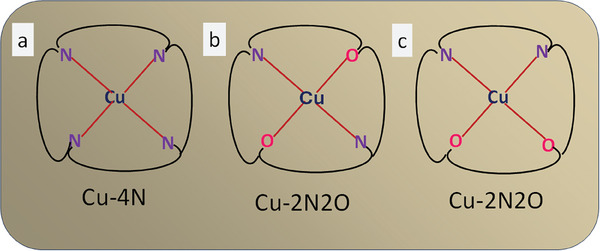
Schematic representation of Cu‐SAC in chelating environments with various symmetry. a) Cu‐SAC in four nitrogen‐coordinated symmetrical environments. b) Cu‐SAC (*trans* symmetry) in a two nitrogen‐ and two oxygen‐coordinated environment. c) Cu‐SAC (*cis* symmetry) in a two nitrogen‐ and two oxygen‐coordinated environment.

It was long speculated that coordination symmetry (CS) has an effect on NRA activity. Cheng et al.^[^ ^60^ ^]^ elegantly demonstrated the effect of CS of the TNMSAC with Cu as the transition metal for ammonia production from nitrate. They showed that the CS of Cu‐TNMSAC influences the bonding, interaction, mechanism, and yield of the ammonia. To test their hypothesis, they selected three systems with different CS. One system was fully symmetrical and others with symmetry breaking.. In the first system, Cu‐TNMSAC is coordinated by four nitrogen atoms in the graphene nanosheet matrix (**Figure** **3**a). The Cu‐TNMSAC has four identical heteroatom groups, creating a uniform and homogeneous chemical environment. In the second system, Cu‐TNMSAC is coordinated by two nitrogen atoms and two oxygen atoms in the graphene nanosheet matrix. The heteroatoms are in *trans* position to each other (Figure 3b). The third system has Cu‐TNMSAC coordinated by two nitrogen atoms and two oxygen atoms in the graphene nanosheet matrix. The heteroatoms are in *cis* position to each other (Figure 3c). These two systems exhibit a breaking the CS symmetry. Figure 3a–c show the schematic representation of these three systems.

The concept of CS revolved around all three systems. At first, Vienna Ab initio Simulation Package (VASP) calculations were conducted on the systems under study to identify the best surface for the adsorption of nitrate and effective reduction into ammonia. Further, the best system post the simulation study was synthesized and experimentally verified for its enhanced activity in the electrochemical production of ammonia. The authors further explained the reason behind choosing a system with two different types of heteroatoms in a *cis/trans* fashion. The *cis* Cu‐2N‐2O‐TNMSAC was chosen because the orientation of the heteroatoms around the Cu‐SAC originates a localized asymmetry due to the difference in chemical environment and interaction among them. This, in turn, produces a regional polarity difference around the surface of the catalyst, which attracts the charged nitrate ions from the electrolyte solution to adsorb onto it. The higher the localized asymmetry generated on the surface of the catalyst, the better the adsorption of the nitrate ions and vice versa. Hence, the order of localized polarity in descending order can be written as *cis* Cu–2N–2O‐TNMSAC, *trans* Cu–2N–2O‐TNMSAC, and Cu–4N‐TNMSAC. Simulation studies were carried out on all three systems under study and radial distribution functions (RDFs) were obtained. The RDFs suggested that the system that attracted the maximum amount of nitrate ions toward its surface was *cis* Cu–2N–2O‐TNMSAC. A follow‐up RDF study was conducted on all three systems to analyze the mode of binding of the nitrate ions on the surface of the considered catalyst. An interesting outcome obtained from the RDF was that the attachment of oxygen on the catalyst surface was much more efficient than the attachment of nitrogen on the surface of *cis* Cu–2N–2O‐TNMSAC.

As an element member of the 3d transition metal series, copper has five d orbitals in its outer shell whose energy is influenced by the ligands bonded to it. Due to the difference in chemical environment of the *cis* Cu–2N–2O‐TNMSAC, *trans* Cu–2N–2O‐TNMSAC, and Cu–4N‐TNMSAC, the d orbitals of copper are split in different ways. The orbitals with the highest energy for *cis* Cu–2N–2O‐TNMSAC, *trans* Cu–2N–2O‐TNMSAC, and Cu–N‐TNMSAC are d_x2‐y2_, d_z2_, and d_xz_, respectively. The symmetry object generated by the *cis* Cu–2N–2O‐TNMSAC system matched the pi* orbital symmetry while *trans* Cu–2N–2O‐TNMSAC and Cu–4N‐TNMSAC matched the *sigma* orbital symmetry. This symmetry matching helps the system achieve stability and aids in the reduction of nitrate to ammonia. Following the simulation studies, the inference was obtained that *cis* Cu‐2N‐2O‐TNMSAC proved to be the best of the three systems for the effective adsorption of nitrate to ammonia. Hence, to experimentally verify the simulation results, the authors synthesized *cis* Cu–2N–2O‐TNMSAC. The synthesis was carried out in two steps. The first step consisted of the preparation of Cu precursor. Cupric acetate in tetra hydrofuran was mixed with phenylenediamine and dihydroxy‐benzenedicarboxaldehyde. The final product obtained after the carbonization process was *cis* Cu–2N–2O‐TNMSAC. The *cis* Cu–2N–2O‐TNMSAC was subjected to LSV studies (0–2.5 V) under a control condition with sodium sulfate (without nitrate) and a nitrate reduction condition (sodium sulfate and potassium nitrate). The control LSV curves showed no appreciable loss in current density while the LSV curve corresponding to the nitrate reduction condition showed a detectable decrease in current density. To test the *cis* Cu–2N–2O‐TNMSAC for its industrial‐grade applicability, a continuous‐flow H‐type cell was constructed for conducting electrolysis experiments. The electrolyte solutions were refilled after 60 continuous hours of electrolysis and spectrophotometrically analyzed. The catalyst was tested for long‐term stability by continuous electrolysis experiments for 2000 h. The system maintained a current density if 360 mA cm^−2^, attesting its excellent attribute of long‐term stability. The performance and stability of *cis* Cu–2N–2O‐TNMSAC was compared to that of other established nitrate‐reducing catalysts like iron single atoms, rhodium single atoms on copper, iron single atoms on molybdenum sulfide, copper chloride on titanium dioxide, titanium dioxide, copper nanowires on copper oxide, cobalt oxide on titanium, palladium copper on tin oxide, copper–silver thin film, copper–palladium thin film, silver–platinum thin film, rhodium–copper nano cubes, ruthenium nanoclusters, Cu cylinder, Fe plate, Pd–Zr–MOF *etc*. *Cis* Cu–2N–2O‐TNMSAC was found to be at par with all the previously established catalysts used for nitrate reduction to ammonia. No other catalyst reported lately had such exceptional stability of 2000 h of long electrolysis sustainability, nor did they exhibit high selectivity and ammonia production yield at the same time. Thus, after the comparison, it was found that *cis* Cu–2N–2O‐TNMSAC showed industrial‐grade standards for the reduction of nitrate ions to ammonia gas and could be successfully explored for the industrial production of ammonia for meeting large energy demands and addressing the global crisis. The rate of ammonia production was calculated to be 28.7 mg h^−1^ cm^−2^ with a selectivity of 80% and FE of 70%.

The mechanism of nitrate reduction is driven by the lower energy barrier of the formed complex (activated) available for accepting electrons from the highest occupied molecular orbitals (HOMO) of the Cu d orbitals. The formed complexes, which are anti‐bonding upon accepting the electrons, show a decrease in N─O bond strength. This is due to the enhancement in the bond length. This results in a facile cleavage of the N─O bond, which further facilitates the overall reduction process of nitrate ions to ammonia. The adsorption of nitrate ions on all three systems heralds the catalysis process, which is followed by the reduction of the two consecutive oxygen atoms in a spontaneous manner. The next step is the addition of a proton to the NO activated species. This step is crucial for the subsequent reduction steps. The energy barrier corresponding to this reduction procedure is different for all three systems. Arranging the energy barrier required to overcome the progress of the reaction in ascending order, we have *cis* Cu–2N–2O‐TNMSAC, *trans* Cu–2N–2O‐TNMSAC, and Cu–4N‐TNMSAC. Hence, *cis* Cu–2N–2O‐TNMSAC requires the least energy to overcome the energy barrier and thus proceeds seamlessly with the reduction process. The reason behind the difference in the energy barrier for the three systems lies in the fact that for the *cis* Cu–2N–2O‐TNMSAC system there is a pi* symmetry‐matched activated energy state, which is readily available for accepting electrons, whereas for *trans* Cu–2N–2O‐TNMSAC and Cu–4N‐TNMSAC a *sigma* symmetry‐matched activated energy state is available. The *pi* *symmetry‐matched activated energy state is lower in energy than the *sigma* energy state. The different energy levels dictate the reaction mechanism of nitrate reduction. The next three steps are the addition of a proton to the activated NOH species to yield NHOH and NH_2_OH followed by the release of NH_3_ gas.

The silver‐based catalyst also showed symmetry‐based behavior. The excellent electrocatalytic behavior of ammonia was shown using *trans* nitrogen and oxygen coordinating Ag‐SAC.^[^ ^61^ ^]^ The concept of electrocatalytic action is dictated by the presence of *trans* coordination of the hetero ligands surrounding Ag‐SAC. The hetero ligands present a chemical asymmetric environment around the Ag‐SAC, thus facilitating the efficient adsorption of nitrate ions. The asymmetry also favors preferentially higher adsorption activity of nitrate ions over other intermediates like NO_2_ and NH_2_OH. This behavior, in turn, is quite helpful in the liberation of ammonia gas at the end of the reaction. The Ag‐SAC displays 98% energy efficiency, distinctly pointing out the fact that all the energy is consumed to produce ammonia from nitrate with only 2% energy loss, which is quite considerable. Looking into the conversion mechanism suggests that the *trans* configuration of the chelating ligands not only aids in the preferential activation of the N–O bond cleavage of the nitrate ions but also assists in the suppression of the hydrogen evaluation reaction in the electrolyte medium. Hydrogen evolution reaction (HER) continues to challenge the overall productivity of ammonia in the electrochemical synthetic strategy because of its competitiveness with the process of nitrate adsorption on the surface of the catalyst. However, Ag‐SAC preferentially adsorbs the nitrate ions over hydrogen ions, classically eliminating the chances of excess waste product formation right from the initial step. The energy efficiency of the Ag‐SAC is high, which also suggests high selectivity toward ammonia gas formation. This approach of *trans* hetero chelating ligands has proven to be the best type of configuration while manufacturing Ag‐SAC as they are responsible for a higher amount of ammonia yield of 90 mol h^−1^ g^−1^. Such higher yields point out the fact that these types of catalysts can be used for the large‐scale production of ammonia in industries, successfully substituting the Haber‐Bosch process.

### 2.4 The Effect of Electrochemical Potential on Single Atom Catalyst Stability

**Figure 4:**
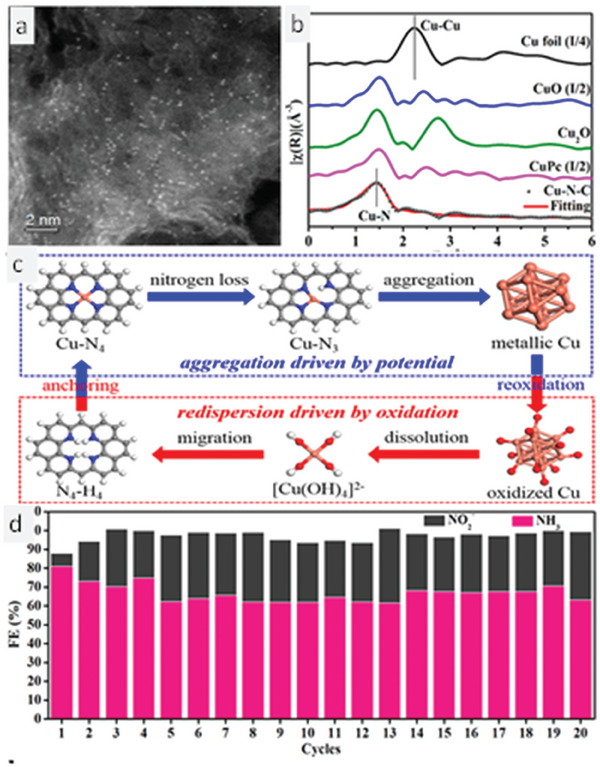
Potential‐dependent catalytic evaluation of Cu‐TNMSAC: characterization, potential‐driven structural switching, and catalytic stability. a) HAADF‐STEM micrograph of Cu‐TNMSAC. b) Comparative XANES results of the collected Cu‐TNMSAC at various potentials showing the Cu─Cu bond length for various samples. c) Potential‐induced changes in the Cu‐TNMSAC structure and its reversible switching mechanism reveal the various intermediate transition structures of Cu‐TNMSAC. d) Cycling stability of Cu‐TNMSAC for 20 consecutive electrocatalytic reduction cycles yielding ammonia. Reprinted with permission from ref. [62]. Copyright 2022 American Chemical Society.

The applied electrochemical potential plays a pivotal role in effective electrochemical NRA. But the fact that is often overlooked is the stability of the catalyst throughout the potential window. One such work, which beautifully portrays the consequence of potential applied on the stability of the TNMSAC and its overall consequence for the NRA reaction is presented by Yang et al.^[^ ^62^ ^]^ These authors synthesized Cu‐TNMSAC in a two‐step method. Silica was used as a hard template for the anchoring of the precursor matrix. 2,6‐diamino pyridine was mixed with sodium hydroxide, and ammonium persulfate was added in a drop‐wise fashion under stirring conditions for 12 h in an ice bath maintained at 4 °C. The precipitate obtained after the reaction was recovered, filtered, and dried. The silica particles that were used as a template were further removed by acid etching with hydrogen fluoride. Following the removal of silica, the sample was mixed with copper nitrate in a mixture containing ethanol as a solvent, and sonicated for 30 min. The reaction mixture was further stirred for 12 h and subjected to a carbonizing environment at 500 °C, finally yielding Cu‐TNMSAC. Cu‐TNMSAC was characterized using the high angle annular dark field scanning tunneling electron microscope (HAADF‐STEM) imaging mode in STEM as shown in **Figure** **4**a. The synthesized Cu‐TNMSAC is coordinated by four nitrogen atoms. The overall activity exhibited by the catalyst lies in the electroactive site of the Cu‐TNMSAC. Unfortunately, few studies discuss the aspect of stability retention and the reconstruction of electroactive sites when exposed to different applied potentials. Therefore, a potential‐depended study was carried out to reveal the NRA catalytic attributes at various applied potentials. The reconstruction may lead to an enhancement or decline in the overall activity of the NRA reaction. The working potential was been limited to a potential window of 0 to −1 V versus RHE.

The authors demonstrated the direct conversion of nitrogen to ammonia gas using a fabricated catalyst, which could offer a sustainable solution to the imbalanced nitrogen cycle. However, this approach requires a lot of energy, so they proposed a plasma‐assisted conversion of nitrogen gas to water‐soluble oxides of nitrogen. These oxides of nitrogen (ions) were then subjected to NRA catalytic conditions (0 to −1 V). The electrolyte for the NRA was a mixture of KOH and KNO_3_ while the control experiments were done without KNO_3_. The main objective of the experiments was to examine changes in the stability, structure, and conformation of the catalyst during the reaction under different potentials. Operando *X‐ray* absorption spectroscopy was used to determine in situ structural changes in the Cu‐TNMSAC catalyst. As the reaction proceeded, the concentration of nitrate ions decreased following roughly first‐order rate kinetics. The ammonia output from the experimental setup increased gradually with the increase of applied potential. The operando X‐ray absorption near edge structure (XANES) results revealed that the structure of the Cu‐TNMSAC catalyst underwent significant changes. Figure 4b shows the comparative XANES results of the Cu‐TNMSAC collected at various potentials during the long‐term electrolysis experiments. The XANES results also show the results of copper foil, copper oxide, and cupric oxide particles along with the Cu‐TNMSAC at different potentials (0, −0.2, −0.4, −0.6, −0.8, and −1.0 V). The initial XANES results did not match with the XANES results obtained at a higher potential, indicating that the Cu‐TNMSAC no longer existed as single atoms but underwent changes to some other form of copper. Accordingly, the Cu single atoms were stabilized by a set of four homogeneously bonded nitrogen atoms (Cu–N_4_). When the potential increased the single atom coordination underwent a restructuring to form a structure that is no longer coordinated to four different nitrogen atoms but reduces to three nitrogen atoms. Because of the decrease in the coordination of nitrogen atoms, the system upholding the Cu single atoms becomes quite unstable and, hence, moves toward a process of aggregation or agglomeration. Thus, with a small increase in the potential, the Cu–N_3_ further degrades and aggregates to form nanoparticles. At this moment, the readers might assume that the fabricated SAC is not effective enough as it undergoes decomposition into different nanoparticles and might also reduce the overall yield of the ammonia at the end of the reaction. On the contrary, the restructured catalyst exhibited boosted overall performance on ammonia yield. The restructuring of the atoms aids in the overall NRA reaction. There also exists an effect on the structural changes occurring on the surface of the Cu‐TNMSAC. The authors revealed that after the single atoms aggregated into nanoparticles, were exposed to the environment, and inserted back into the electrolyte solution, they underwent oxidation to copper hydroxide and dissolution followed by the attachment of the Cu atoms in the empty sockets of the four N atoms‐coordinated matrix, thus yielding back the Cu‐TNMSAC. This phenomenon (Figure 4c) can be quite misleading as the catalyst obtained in the post‐experimental conditions is the same as in the pre‐experimental conditions. Looking into the reaction superficially, it can be inferred that the catalyst is quite stable and does not undergo any noticeable changes in the structure or function even when higher potential is applied to it. The operando XANES results helped provide insight into the changes in the structure of the Cu‐TNMSAC, revealing the distance between the Cu atoms changes for varying potentials, giving strong proof of the various transitions occurring on the Cu‐TNMSAC.

A superficial analysis of the reaction might suggest that the catalyst is stable and does not change its structure or function significantly even at higher potentials. However, the operando XANES results provided insight into the structural changes of the Cu‐TNMSAC, which showed that the distance between the Cu atoms varied for different potentials, indicating the various transitions occurring on the Cu‐TNMSAC. Regarding ammonia production, the yield increased with the potential. The restructuring enhanced the ammonia yield. In other words, the aggregated Cu nanoparticles produced more ammonia than the Cu‐TNMSAC. The yield at a high potential of −1 V was 4.5 mg cm^−2^ h^−1^. The catalyst also maintained a high FE of 85%. The Cu‐TNMSAC underwent a long‐term stability test, which showed that it retained 70% FE even after 20 catalytic cycles (Figure 4d). This result contradicted the misconception that the Cu‐single atoms were less efficient than the Cu nanoparticles at higher potential.^[^ ^54^ ^]^ The efficiency of large particles and small clusters was also compared and it was found that the former had lower faradaic efficiency than the latter at a potential of −0.6 V. The reaction mechanism on the surface of the Cu–N_3_ did not favor the formation of ammonia. The reaction was hindered by the inability of the Cu–N_3_ to reduce activated NO_2_ to NO and then to ammonia. On the contrary, the Cu cluster facilitated the electrochemical production of ammonia. The Cu clusters helped in the effective adsorption of nitrate ions and the formation of activated NO species from NO_3_^−^ ions. The next steps were the protonation of the activated NO to HNO and the addition of another proton to form an activated NHOH species. From this step, the mechanism could follow two different pathways, both leading to ammonia. The first pathway was the direct protonation of the activated NHOH to form NH_2_OH species. The second pathway was the formation of activated NH species with the elimination of water from the activated NHOH species. The final step was the release of ammonia gas.

### 2.5 The Interaction of Cocatalyst with Transition Metal Single Atom Catalyst

Another interesting aspect of TNMSAC is the role of the cocatalyst and its interaction with the transition metal single atoms to improve catalytic activity for NRA. The cocatalyst‐based studies are novel and provide detailed information about the mechanism of action. Liu et al. investigated the concept of cocatalyst by using two different transition metals, rhodium and copper, in a single system. They have synthesized copper nanowires and decorated them with single atoms of rhodium on the surface.^[^ ^47^ ^]^ The Rh‐TNMSAC@Cu was synthesized in a three‐step reaction. Initially, the copper oxide nanowires were fabricated and used as substrates, and then reduced electrochemically to yield Cu nanowires. Finally, the Cu nanowires were decorated with rhodium single atoms. The decoration occurred due to the ion exchange between the Cu substrate and the rhodium from the electrolyte solution. The Rh‐TNMSAC@Cu was characterized by the scanning electron microscope (SEM) and HAADF‐STEM. The presence of rhodium single atoms was further determined from the distance between two rhodium atoms (less than 0.2 nm) from the EXAFS spectra. The authors were interested in studying the effect of rhodium single atoms on the overall NRA activity of the Cu nanowires. Hence, to elaborately examine the aforementioned effect, they conducted LSV studies to look into the aspect of generation of ammonia from the catalyst. For LSV measurements, two different electrolyte systems were selected: (i) sodium sulfate and (ii) a mixture of sodium sulfate and potassium nitrate. Three different catalyst systems were chosen: Rh‐TNMSAC@Cu with a distribution of 0.3%, 0.6% rhodium single atoms, and 12.5% loading of rhodium nanoparticles on Cu nanowires. The LSV curves reveal that Rh‐TNMSAC@Cu with 0.6% single atom loading shows the highest reduction in current density, indicating good ammonia yield activity. Rh‐TNMSAC@Cu with 0.3% single atom loading, shows low performance in comparison to the 12.5% loading of rhodium nanoparticles on Cu nanowires. Hence, this study clears the fact that the mere presence of single atoms does not boost the performance of a catalyst, rather, its composition and distribution are equally important. A comparative study was carried out to compare the faradaic efficiency of the Rh nanoparticle, Rh‐TNMSAC@Cu, and Cu nanowires. It was found that Rh nanoparticles displayed the least faradaic efficiency over a range of potential (0 to −0.3 V vs RHE). The best faradaic efficiency was displayed by Rh‐TNMSAC@Cu followed by pristine Cu nanowires. The results provide a strong indication toward the enhancement of the NRA activity of Cu nanowires upon the addition of Rh single atoms.

The density functional theory (DFT) study of the nitrate reduction mechanism was supported by the electron paramagnetic resonance (EPR) results of the electrolyte solution after the NRA reaction. TheEPRresults showed that the hydrogen radical was stable in the case of rhodium nanoparticles, whereas it was hardly detectable for the Rh‐TNMSAC@Cu and pristine Cu nanowires. This indicated that the hydrogen radical was adsorbed by the rhodium single atoms and Cu nanowire system. Pristine rhodium nanoparticles were inactive for NRA catalysis because they could not attract the nitrate ions on the surface, instead, they were more active in the reduction of water to form activated hydrogen species. Therefore, they were used as cocatalysts to utilize their activity for activated hydrogen species generation and to support the overall NRA process. The overall reduction of nitrate to ammonia involved the reduction of NO_3_ to NO activated species in three consecutive steps, which was thermodynamically favorable because the process had negative Gibbs free energy. The fourth step was crucial as the subsequent reduction depended on it and it was the rate‐limiting step. The energy states for Cu‐ and Rh‐TNMSAC@Cu are shown in light blue and dark blue colors, respectively. The energy states showed that the first three steps of NRA were similar for both pristine Cu nanowires and Rh‐TNMSAC@Cu. The rate‐limiting step was the fourth step of the reaction, where a proton was transferred to the activated NO species. This step was difficult for the pristine Cu nanowires as the Gibbs free energy for the process was higher. Therefore, the hydrogenation step was slower, which affected the faradaic efficiency and yield of ammonia. For the Rh‐TNMSAC@Cu, the ∆G value for the hydrogenation process was more negative than the formation of activated NO species. This meant that the hydrogenation process was highly favorable and fast as it involved the transfer of the in situ generated hydrogen without any electron involvement along the catalyst surface. From the ∆G value for the hydrogenation process for the two systems, it could be inferred that the rhodium single atoms lowered the energy levels and facilitated the transfer of activated hydrogen to the activated NO to form NOH activated species. The rhodium single atoms also reduced the energy levels of the subsequent hydrogenation steps, which resulted in the easy formation and desorption of ammonia gas. The rhodium single atoms as cocatalyst also helped in achieving a good ammonia yield even at lower potentials. At higher potentials, the Cu‐based catalyst systems generally produced hydrogen as a byproduct, as they were active for HER in that range. Adding the rhodium single atoms to copper solved the problem of low selectivity of ammonia generation from nitrate ions at a lower potential. This study opened a window of possibilities for exploring a series of transition metals active for HER and their synergistic effect on NRA activity.

### 2.6 The Effect of Dual Single Atoms on NRA Conversion

**Figure 5:**
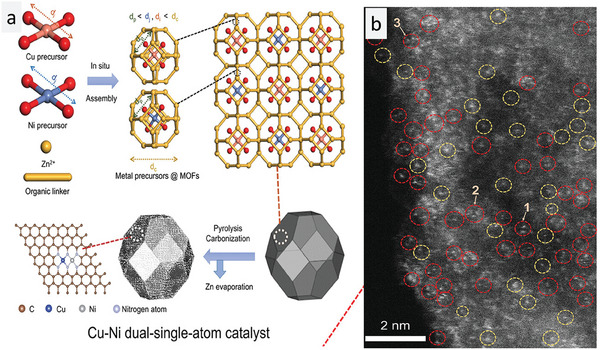
Synthesis and imaging of Cu–Ni dual atom TNMSAC. a) Synthetic process for the preparation of Cu–Ni dual atom TNMSAC. b) TEM micrograph of Cu–Ni dual atom TNMSAC with Cu and Ni single atoms shown in dotted circles.Reprinted with permission from ref. [63]. Copyright 2023 Wiley‐VCH GmbH.

We already know the efficient NRA activity of SAC, but the synergistic effect of a hetero single atom next to the single atom is still unclear. Wang et al. explored this concept by introducing dual single atoms (DSAs) to prevent the aggregation of single atoms.^[^ ^63^ ^]^ In a DSA system, two different single metal atoms are placed next to each other without any direct bond between the two SACs. The chelating groups on the two adjacent groups stabilize them so effectively that they eliminate any chance of aggregation, making dual single atom catalyst (DSAC) systems more stable than the SAC system. The orientation of the metals allows the free migration of electrons from one single atom to another due to their mismatched polarity and electronic distributions. In this work, the DSA consisted of Ni and Cu. The system was made heterogeneous such that Ni single atoms had a higher affinity for the adsorption of activated H and other important intermediates, whereas Cu single atoms preferentially adsorbed the activated N species. This combination was ideal for highly selective product formation as it prevented the formation of N_2_ gas as a by‐product and helped in the hydrogenation of NO activated species. The DSA was synthesized using zeolitic imidazolate as a base matrix and the mixed with Cu and Ni precursors. After the metal ions were incorporated into the zeolitic imidazolate framework (ZIF)framework, they were carbonized to obtain the DSA system. The arrangement of the Ni and Cu single atoms in the carbon framework and its synthetic process are shown in **Figure** **5**a. The TEM micrograph was used in the imaging of Cu and Ni dual atoms. For better clarity, the dual atoms have been encircled in yellow and red color (Figure 5b). Comparative LSV measurements were performed on Cu‐TNMSAC, Ni‐TNMSAC, and Cu–Ni DSA. As predicted, the DSA system was the best catalyst, with the maximum drop in the current density. Among Cu and Ni single atoms, the former system had better ammonia production than the latter. The FE of Cu–Ni DSA at −0.7 V versus RHE was 95%. A cyclic stability result showed 90% efficiency even after 20 cycles, making the catalyst highly desirable for industrial applications. The high yields of ammonia were due to the synergistic mechanism of work between Cu and Ni in the easy reduction reaction and the prevention of unnecessary by‐products.

Recently, Wan et al.^[^ ^64^ ^]^ hypothesized a unique way of material design in catalysis and later also proved it with experimental data about TNMSAC. They proposed that a mere strategic design of the catalyst with two different single atoms could help resolve the sluggish reaction kinetics of the electrochemical conversion of nitrate to ammonia. The authors encountered that, generally, single atoms of transition metals could be designed in such a way as to deal with either the conversion of nitrite from nitrate ion or the conversion of nitrite to ammonia. While most of the reported SACs dealt with the former reaction, the latter was taken care of mostly from the hydrogen generated from the water‐splitting reaction, thus resulting in the reduction of nitrite to ammonia at higher potentials. However, the disadvantage of this approach lies in the fact that at higher working potential values there occurs competition between two reactions, *i.e*., between the conversion of nitrite to ammonia and the evolution of hydrogen from water. Hence, the overall faradaic efficiency decreases because of the formation of side products. To solve this issue, they designed a catalyst comprising two different single atoms of Mo and Fe. The Mo single atoms and Fe single atoms have different functions concerning the catalytic process. Mo single atoms preferentially convert nitrate to nitrite for the NRA catalysis and the Fe single atoms preferentially catalyze the reduction reaction of nitrite to ammonia. Regarding the dual single atom Mo–Fe‐TNMSAC, the single atoms work synergistically in cooperation with each other to generate ammonia. Because of the unique design and working synergy, a high faradaic efficiency of 94% was reported with an ammonia yield of 13.56 mg cm^−2^ h^−1^.

### 2.7 The Effect of Diatoms on NRA Catalytic Activity

**Figure 6:**
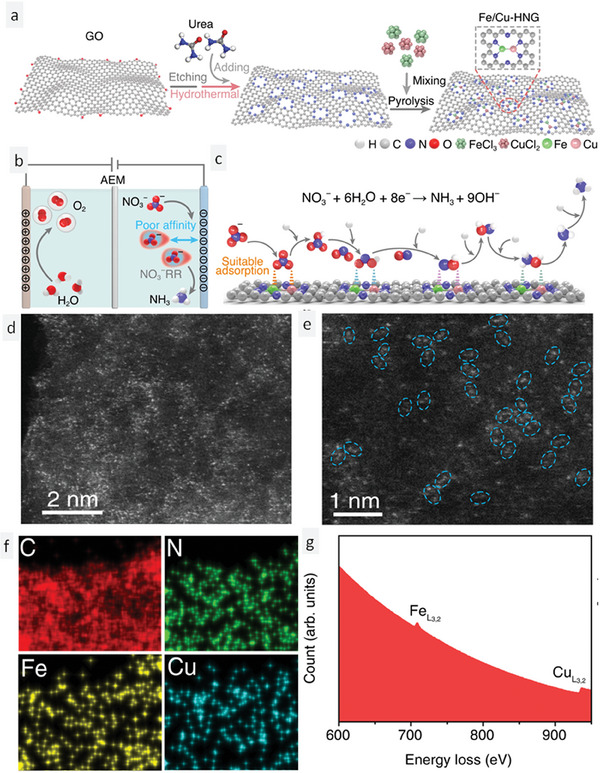
Experimental insights into dual atom Fe–Cu‐TNMSAC for efficient electrochemical NRA catalysis. a) Schematic representation of the synthesis of Fe–Cu‐TNMSAC. b) H‐type cell for ammonia generation. c) Pictorial representation of the catalytic conversion of NRA reduction at the cathodic part. d,e) HAADF‐STEM micrographs of the dual atoms at 2 nm and 1 nm scale, respectively. f) EDX micrograph of Fe–Cu‐TNMSAC in an NHGS matrix. g) EELS spectra of Fe–Cu‐TNMSAC. Reprinted with permission from ref. [65]. Copyright 2023 Springer Nature.

After discussing single atoms in detail, another interesting concept is diatoms. This raises the question: are they the same as single atoms or dual atoms, or do they have unique attributes that are different from the single/dual atoms? Diatoms, as the name implies, are composed of two connected single atoms. Zhang et al.^[^ ^65^ ^]^ demonstrated the concept of diatoms and their application in NRA by synthesizing a Fe–Cu‐TNMSAC and testing its efficiency for electrochemical ammonia production. They synthesized the dual atom TNMSAC on a hollow graphene sheet with ample induced porosity. The graphene sheet was obtained from the modified Hummers method^[^ ^66^ ^]^ and oxidized with concentrated nitric acid to obtain a nitrogen‐doped hollow graphene sheet (NHGS). The NHGS was mixed with copper and iron precursor salts, and subjected to solvothermal treatment in an autoclave to obtain a hydrogel, which on additional carbonization conditions yielded a powder comprising dual atoms of Fe–Cu TNMSAC in an NHGS matrix. A schematic representation of the synthesis process is shown in **Figure** **6**a. The setup used for the NRA reduction reaction in an H‐type cell is shown in Figure 6b. The setup consists of two components, the cathodic part where nitrate ions are reduced to ammonia and the anodic part where water is converted to oxygen gas. The pictorial representation of the catalytic conversion of NRA reduction at the cathodic part is shown in Figure 6c. The morphology of the dual atoms was characterized using HAADF‐STEM micrographs. Figure 6d,e show the HAADF‐STEM micrographs of the Fe–Cu dual atoms at 2 nm and 1 nm scale, respectively. The dotted oval structures in Figure 6e reveal the arrangement of dual atoms of Cu and Fe atoms. Figure 6f shows the EDX micrograph of the Fe–Cu‐TNMSAC in an NHGS matrix, distinctly ascertaining the presence of Cu, Fe, N, and C in the sample. The composition of the Fe–Cu‐TNMSAC was examined using EELS (Electron Energy Loss Spectroscopy). EELS revealed the simultaneous presence of Cu and Fe through the energy loss spectrum (Figure 6g).

**Figure 7:**
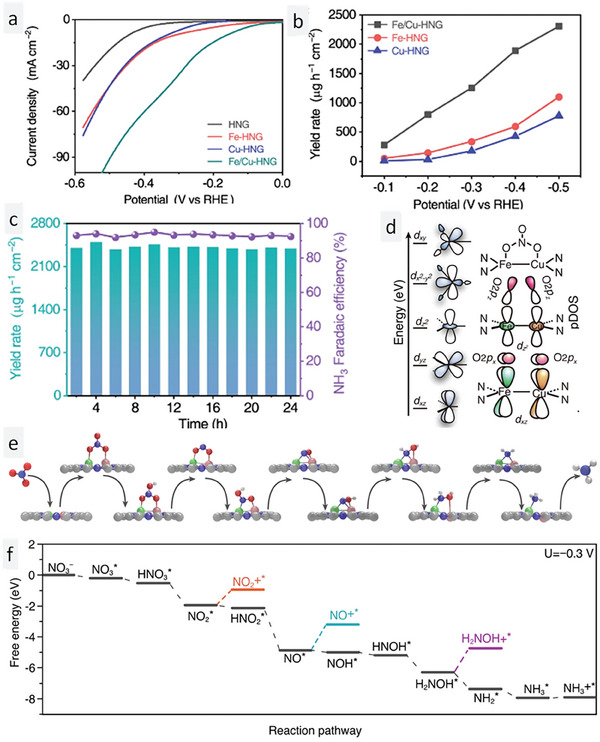
Experimental and theoretical evaluation of dual atomic Cu–Fe‐TNMSAC for the electrocatalytic NRA reaction. a) LSV curves of NHGS, Cu‐TNMSAC, Fe‐TNMSAC, and Cu–Fe‐TNMSAC. b) Ammonia yield rate of Cu‐TNMSAC, Fe‐TNMSAC, and Cu–Fe‐TNMSAC. c) Cycling stability of Fe–Cu‐TNMSAC dual atoms catalyst. d) Orbital diagram showing the interaction between nitrate and Fe–Cu dual atoms. e) Mechanism of reduction of NRA on the surface of the Fe–Cu dual atom centers. f) DFT‐calculated variation of free energy and reaction pathways involving various intermediates in the formation of ammonia. Reprinted with permission from ref. [65]. Copyright 2023 Springer Nature.

The inspiration behind dual atoms revolves around the induction of heterogeneity, which results in the enhanced accumulation of nitrate ions on the catalytic surface. The higher the polarity induced by the active centers, the better the adsorption of nitrate ions. The ammonia yield has a direct relationship with the adsorption of nitrate ions on the surface of the catalyst. It can be seen that the interaction between the p orbitals of the oxygen atoms of the nitrate and the d orbitals of the Cu–Fe dual atom are responsible for decreasing the energy threshold for the adsorption of ions on the catalyst surface. Additionally, the presence of a *hetero* environment projected by the diatoms seamlessly activates the N─O bond for the efficient bond‐breaking and reduction required for the progress of the reaction for producing ammonia. To ascertain the efficiency of the Fe–Cu‐TNMSAC dual atoms, three other systems (NHGS, Cu‐TNMSAC, and Fe‐TNMSAC) were chosen to provide a comparative analysis using LSV measurements (**Figure** **7**a). The curves corresponding to the Fe–Cu‐TNMSAC dual atoms show better reduction activity of nitrate in comparison to the Cu or Fe single atom systems. Hence, this observation proves that the dual atom configuration is much more effective in enhancing ammonia production compared to single atoms. An additional LSV study was also conducted to realize the competing effects of HER in the NRA catalysis reaction. It was found that the Fe–Cu‐TNMSAC dual atoms catalyst was highly selective for NRA and did not show active attributes in the HER potential window. Moreover, the yield rate was compared for all three systems and the best yield was exhibited by the Fe–Cu‐TNMSAC dual atoms catalyst (Figure 7b). Even after 24 h long‐term electrolysis, a high FE of 90% was sustained by the dual atoms (Figure 7c). The dual atoms were characterized after 24 h of electrolysis with HAADF‐STEM and it was observed that the dual atoms were distributed in the catalyst with few alterations.

The mechanism of nitrate reduction was realized with DFT calculations and differential electrochemical mass spectrometry (DEMS) analysis of intermediates. Because of the mixing of the p and d orbitals of oxygen atoms of nitrate and the Cu–Fe dual atoms, there was an appreciable decrease in the adsorption energy, as a result, nitrate ions were adsorbed on the catalyst surface before proceeding for reduction (Figure 7d). Additionally, the authors found from their simulation studies that the extent of nitrate ions adsorbed on the surface of the catalyst varies greatly when compared with the adsorption of other intermediates like NO_2_H and NO_2_. This again highlights the fact that the intermediates can be easily desorbed, enabling the efficient reduction of the intermediate species to form ammonia gas. The mechanism of reduction of NRA on the surface of the Fe–Cu dual atom centers is shown in Figure 7e. The DFT‐calculated variation of free energy and the reaction pathway involving various intermediates in the formation of ammonia are illustrated in Figure 7f. Hence, this work proved through both experimental and theoretical approaches that dual atoms were more effective than single atoms for NRA catalysis.

Because we have already discussed single and diatomic catalysts, it would be especially interesting to understand the difference in their catalytic activity. As far as structure is considered, dual atomic catalysts differ from single atom catalysts in that there are two different single atoms adjacent to each other without any kind of direct bridging bond between them. The surrounding chelating groups stabilize the single atoms and prevent them from aggregating. Catalytically, dual single atoms are more stable than single atoms because single atoms always have a high chance of aggregation at extreme experimental conditions. Hence, dual single atom catalysts show better catalytic performance in terms of cyclic stability. Another difference in the catalytic performance of both the mentioned catalysts is that the dual single atoms show excellent electron migration from one single atom to another. As a consequence of better delocalization of charges (asymmetrical polarization), the dual single atoms show superior energy efficiency and electrocatalytic reduction performance of nitrate than single atoms. The dual single atoms often work in synergy mode between the heteroatoms, which is in contrast to single atom catalysts. When Ni and Cu single atoms were arranged adjacent to each other, Ni atoms showed a strong affinity for hydrogen while Cu atoms had more affinity toward nitrate.^[^ ^65^ ^]^ Therefore, when working together in a synergistic mode, the intermediate N–OH is produced due to localized interaction and yields ammonia selectively. This behavior is in contrast to the single atoms where there is always a possibility of side products although in minute quantities. It is claimed that the efficiency of the dual single atoms is about 98–100% because they do not form side products.^[^ ^63^ ^]^

### 2.8 Enhancing the Stability of Single Atoms Catalysts

**Figure 8:**
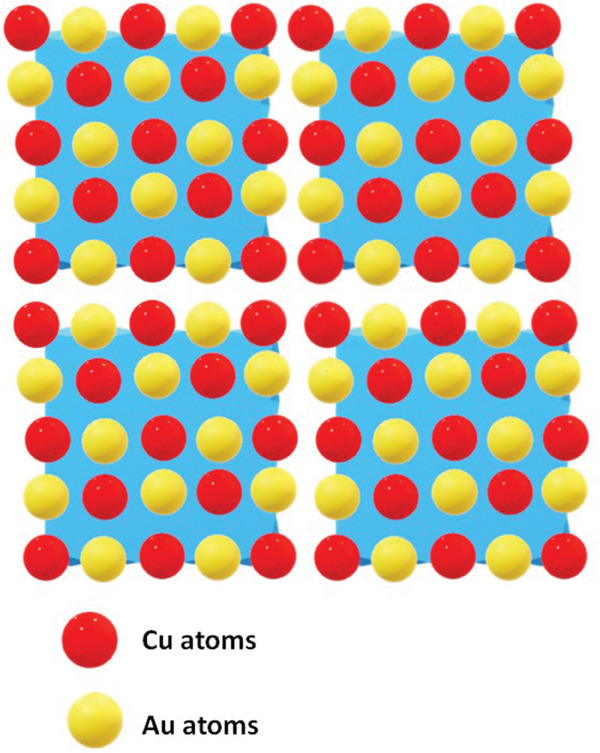
Schematic representation of the arrangement of Au single atoms in Cu nano cubes.

Creating a catalyst with single atom components is challenging. The main problem is the instability of the single atoms, which tend to aggregate into particles to reduce the surface energy. This makes it difficult to design and incorporate them in a catalytic system. Conventional methods for synthesizing multi‐metal atomic crystals involve heating the metallic precursors above 600 °C to allow them to rearrange and form stable structures. However, the temperature often causes the formation of aggregated structures rather than single crystals. Another factor that needs to be considered is the distribution of the single atoms on the catalyst surface. Their loading cannot be too high as that would also promote the aggregation of atoms due to their proximity. Therefore, Gao et al.^[^ ^67^ ^]^ designed a low‐temperature solution‐based synthesis technique for the formation of single atoms of gold arranged in a matrix of copper atoms. They named such precisely arranged structures (SAA) “single atom intermetallic alloys.” They suggested that the arrangement of single gold atoms in the matrix of copper atoms not only solved the issue of instability but also allowed higher loading density of the atoms. They engineered the orientation, density, and strain of the single atoms in the original matrix of copper atoms. All these effects were also analyzed for NRA catalysis. The Au‐TNMSAC@Cu nano cubes were synthesized using Cu nano cubes as hard templates/substrates in two steps. First, Cu nano cubes were synthesized using a Cu precursor and a reducing agent dissolved in a solvent in an inert atmosphere. In the second step, the Cu nano cubes were used as hard templates and chloroauric acid was used as a precursor for gold single atoms. The mixture was aged together, which resulted in an ion exchange reaction between copper and gold, yielding the Au‐TNMSAC@Cu nano cubes. The percentage of gold single atoms was also controlled by the concentration of chloroauric acid in the reaction mixture. A schematic representation of the arrangement of Au single atoms in Cu nano cubes is shown in **Figure** **8**. The synthesized Cu nano cubes and Au‐TNMSAC@Cu nano cubes were characterized using bright‐field TEM micrographs along with their HAADF‐STEM micrographs. The atomic resolution HAADF‐STEM micrographs reveal the atomic‐level arrangement (d spacing) of copper atoms and gold single atoms in a copper matrix. EDX analysis of the prepared Au‐TNMSAC@Cu nano cubes shows the presence of copper atoms and gold atoms.

Among the prepared materials, the as‐prepared Au, Cu nano cubes, Au‐TNMSAC@Cu nano cubes, and ordered Au‐TNMSAC@Cu nano cubes exhibited the best performance by an ordered single atom alloy based on linear sweep voltammetry and chronoamperometric studies. The current density of the ordered SAA showed a drastic change, indicating ammonia production from the reduction of the nitrate ions. An FE as high as 85% was exhibited by the ordered Au‐TNMSAC@Cu nano cubes at a potential of −0.5 V versus RHE. The yield rate of ammonia was also high and at par with most of the recently established literature.

DFT calculation of the electrochemical nitrate reduction was carried out using VASP in order to gain insight into the mechanism of action. The calculations were based on the Cu (100) plane as a previous study showed that the maximum adsorption of the nitrate ions from the solution occurred on the Cu (100) surface.^[^ ^68^ ^]^ The authors compared the nitrate absorption capacity on three different systems: Cu nano cubes, sparsely distributed Au‐TNMSAC@Cu nano cubes, and ordered Au‐TNMSAC@Cu nano cubes. While comparing the adsorption activity of the nitrate ions on the three different surfaces, it was found that they have maximum affinity toward the ordered Au‐TNMSAC@Cu nano cubes followed by pristine Cu nano cubes and, finally, by sparsely distributed Au‐TNMSAC@Cu nano cubes. The adsorption of activated nitrogen atoms on these surfaces follows just the opposite trend, hence making the desorption of ammonia gas after reduction relatively easy. Comparing this behavior in light of the d band theory, it is quite evident that the position of the d band centers influences the adsorption of the nitrate ions. Thus, the d band centers can be arranged in ascending fashion as follows: sparsely distributed Au‐TNMSAC@Cu nano cubes, Cu nano cubes, and ordered Au‐TNMSAC@Cu nano cubes. An additional consequence of the d bond centers also contributes toward the adsorption of the activated nitrogen species. However, the desorption of the ammonia is easier due to the presence of gold atoms on the surface of the catalyst. The gold atoms represent a fully saturated d orbital configuration, which restricts the hybridization of the nitrogen species and results in an overall repulsive action, thus promoting a better yield of ammonia by easily desorbing it from the catalyst surface. Hence, the mechanistic action for ammonia generation from both types of Au‐TNMSAC@Cu nano cubes can be directly related to the concentration of the Au single atoms in the Cu matrix. The higher the concentration of the single gold atoms the better its performance in terms of ammonia yield and vice versa.

## 3 3D‐Printed Single Atom Catalysts

**Figure 9:**
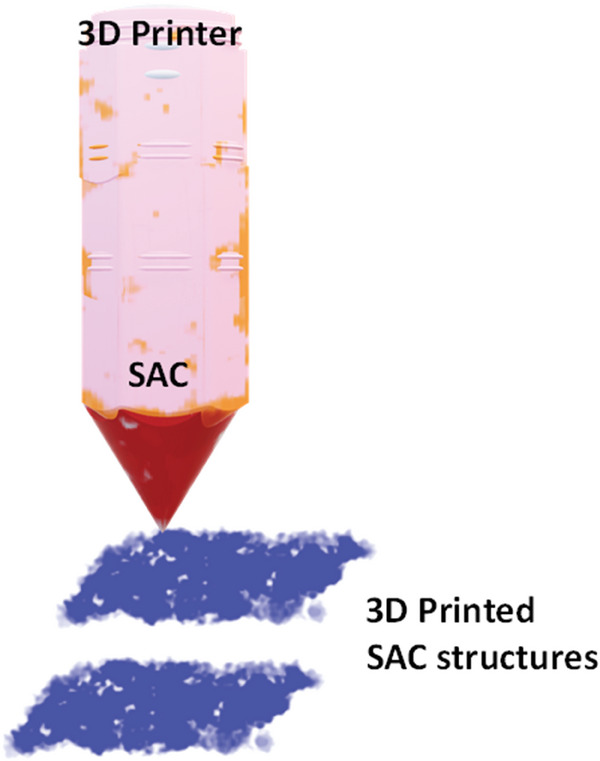
Schematic representation of the 3D printing of TNMSAC structures for the electrocatalytic generation of ammonia from nitrate ions.

Finally, after all the insightful discussions, we have also tried to amalgamate the concept of additive manufacturing in the field of NRA catalysis. Because of its manifold advantages, additive manufacturing has extended to various fields of study and provides a generic solution to many problems like tuned scalability, reproducibility, sustainability, low cost of manufacture etc. Single atoms will always be a challenge because of stability issues, however, what could be a fascinating approach is to eliminate the issue while introducing the novel concept of 3D printing. Lately, Xie et al.^[^ ^69^ ^]^ have presented the concept of generalized transition metal ions‐based single atom 3D printing technology. The printing ink was prepared by taking gelatin as a base matrix and mixing it with a transition metal (TM) precursor (TM acetylacetonate), stabilizers, and photo polymers. With a feed temperature of 30 °C, the printer's photo initiators initiate the polymerization processes. As a result, the photo polymers start crosslinking and forming the gelatin‐based 3D structures along with the incorporation of the transition metal ions. The post‐processing procedure was performed by freeze‐drying the 3D‐printed catalyst to eliminate the water molecules. The structures were further carbonized at a temperature of 700 °C in a tube furnace to finally yield 3D‐printed TNMSAC structures. The authors have fabricated six different transition metals to highlight the versatility of the 3D printing strategy. A schematic representation of the 3D printing of TNMSAC structures is shown in **Figure** **9**.

Two different types of 3D‐printed catalysts (carbon‐based structure and Fe‐TNMSAC structure) were subjected to NRA reduction using LSV studies with KOH and KNO_3_ as electrolytes. As expected, the Fe‐TNMSAC 3D‐printed structures showcased better NRA activity than the carbon‐based 3D‐printed structure. The quantitative analysis of ammonia yield was conducted using an aliquot collected after long‐term electrolysis experiments. The aliquot was subjected to ultra violet (UV)–vis studies after which the yield of ammonia was found to be ≈4.55 µ mol cm^−2^ h^−1^. The cyclic stability test of the Fe‐TNMSAC was conducted by carrying out long‐term electrolysis experiments in an H‐type cell for about 8 h. The ammonia production remained unchanged even after continuous electrolysis cycles revealing the practical application of the catalyst.

The mechanism of NRA catalysis was examined by simulation. The authors claimed that the central transition metal is protected by four chelating nitrogen atoms. The system with TM‐N4 was subjected to DFT calculations. The mechanism according to the DFT calculation can be seen as the adsorption of nitrate ions followed by their reduction to an activated nitrite species. The next step is considered by the authors to be the rate‐determining step, which is the hydrogenation of the activated nitrite species to yield an activated NHO_2_ species. The activated NHO_2_ species further undergoes reduction to form an activated NO species. A second hydrogenation follows to form NHOH in the next step. The remaining three steps are thermodynamically spontaneous and occur with ease, initiated by the conversion of NHOH to activated NH species followed by NH_2_, and finally the elimination of the produced ammonia gas from the reaction setup. This work focuses on a concept that is quite ahead of the other established pieces of literature as it is unique and the first one to focus on the 3D printing of the single atoms for large‐scale and scalable freestanding electrodes for NRA catalysis. This work shall be a guiding light for exploring the polymer‐based 3D printing of single atoms and will also encourage advanced studies leading multi‐material 3D printing or diatomic 3D printing together.

From the above discussions, it is clear that TNMSACs display higher faradaic efficiency than conventional catalysts. Before knowing in detail about the causes behind such an observation, let's discuss some examples of TNMSAC and transition metal nanoparticles in general to obtain a clearer idea. The FE exhibited by Cu‐TNMSAC, Fe‐TNMSAC, Ag‐TNMSAC, Ni‐TNMSAC, Mo‐TNMSAC, and Au‐TNMSAC was 95%, 97%, 98%, 95%, 94%, and 85%, respectively. In contrast, their respective nanoparticles exhibit lower efficiency. For instance, nanoparticles of Cu, Fe, Ag, Ni, Mo, and Au show 80%, 82%, 88%, 79%, 89%, and 84% efficiency, respectively.^[^ ^70^, ^71^, ^72^, ^73^, ^74^, ^75^ ^]^ From the results, we can observe a large difference in the FE of TNMSACs and their corresponding nanoparticles used as active materials in catalysis for NRA conversion. Faradaic efficiency reveals the efficient conversion of nitrate ions to yield ammonia and other by‐products. Low efficiency portrays the occurrence of products in addition to ammonia and the energy utilized in the conversion of nitrate into undesired products. Conversely, a higher efficiency shows that most of the energy is utilized for the production of ammonia and the production of side products is minimal. Hence, improvement in the energy efficiency of TNMSACs can be attributed directly to the selective production of ammonia.

The by‐products of the NRA reaction using TNMSAC are NO_2_, NO, and NH_2_OH. The amount is higher than the other two by‐products in most of the reported literature.^[^ ^76^ ^]^ Although the mentioned products are possible in the TNM catalysis reaction during the NRA reaction, their generated amount is quite low. The efficiency of TNMSACs is usually high: either equal to or higher than 95%. Hence, a large percentage of the total energy is indeed utilized in the production of ammonia gas while a small fraction of the energy is consumed in the production of unwanted side products. There are four major pathways that might be considered for the progression of the NRA reaction. Paths 1–4 are as follows: (1) the conversion of NO_3_^−^ to NH_3_, (2) the conversion of NO_3_^−^ to NO_2_^+^, (3) the conversion of NO_3_^−^ to NO^+^, and (4) the conversion of NO_3_^−^ to NH_2_–OH. Considering the aspect of selectivity of TNMSACs, they show an exclusively high progression of the reaction following path (1), resulting in the production of ammonia. Path (1) is selectively chosen depending on the negative value of the Gibbs free energy because the thermodynamics of the reaction is favored by the most negative value of Gibbs free energy. Hence, of the four above‐mentioned pathways, the pathway resulting in ammonia has the most negative Gibbs energy. These values were ascertained from DFT calculations and crosschecked experimentally.^[^ ^71^, ^73^, ^74^ ^]^ In comparison to conventional catalysts composed of multiple atoms, single atom catalysts show higher selectivity because the reactions and the interactions of the catalyst with the nitrate can be controlled. In contrast, conventional catalysts show lower energy efficiency as most of the applied energy is wasted in uncontrolled reactions, yielding many by‐products and nitrogen gas.

## 4 Conclusions and Future Outlook

This review elucidates the singular behavior of SACs for NRA catalysis from various perspectives. However, each work discussed here offers a discrete package of information about NRA catalysis by single atoms. First, most of the studies have neglected to elucidate the actual distribution of single atoms in the covalent matrix. For instance, how many single atoms are present per area of the matrix, are they uniformly distributed, and what is the effect on the overall catalytic activity if the number of atoms changes? This points to the second pivotal parameter that was absent in most of the studies, *i.e*., ensembles. The concept of ensembles depicts the collective catalytic activity emanating from the single atoms. In a very intriguing review, Guo et al. meticulously described the ensemble effect on catalysis.^[^ ^77^ ^]^ Hence, expressing NRA catalytic activity from the ensemble point of view would provide a pristine picture of the presence, distribution, interaction, and activity of single atoms in the SAC. Third, most studies have overlooked the potential‐ or stress‐induced displacement/alterations of single atoms. To observe whether the atoms are intact in their places and stable enough to carry out catalysis would be an engrossing study to undertake. Another impediment is translation of the SAC concept from the lab to industry. These studies have mostly focused on long‐cycling (20 h), however, to transfer a novel SAC to industry for producing ammonia, parameters such as extensively long cycling for more than 10 000 h and scalability of the synthetic methods for producing, it must be given high priority. Finally, the topic that is given the least attention is the cracking down of the produced NH_3_ gas into H_2_ and N_2_. Ammonia has its own set of applications, but the concept of green energy from ammonia cannot be resolved unless we crack it and extract the hydrogen gas for energy needs. Recently, SAC ensembles have been utilized for cracking ammonia into clean hydrogen. Hence, taking inspiration from this work, a customized setup can be designed composed of SAC first to convert the nitrate into ammonia and then further channelize the output gas through a separate catalytic bed for cracking it to hydrogen gas. This kind of setup can also be seen as a futuristic approach toward sustainable energy generation by converting nitrate‐polluted water to hydrogen directly in two SAC catalytic reactor units.

We identify several areas where more attention is needed:

*Theoretical Insights and Reaction Mechanisms*. More theoretical insights should be provided to enhance the stability of the catalyst. In most reports, DFT calculations have been included for the NRA mechanism, however, additional simulation results concerning the electron density and stability of the catalyst are often overlooked. Enhancing the stability of the catalyst should be given more priority. For example, the introduction of matrix stabilization, better carbon frameworks for stabilization, heteroatoms for better chelation and stability, different hetero single atoms, and other backbones (matrix) like phthalocyanines and similar organic moieties for better stabilization should be investigated. Most literature focuses on the catalyst without giving much attention to the mechanism of action of NRA catalysis. A huge gap is prevalent in the basic understanding of the reaction mechanism of NRA conversion. Hence, this hinders progress in the field of research and development of designing new materials for NRA. Similarly, the catalytic selectivity is conveyed in the manuscripts without giving much attention to the proper mechanism of progression of the reactants to products. Detailed conversion mechanisms forming a product and their associated energies would provide more insight into the performance of the catalyst. Providing mechanistic attributes would further enhance the quality of research in the field of SAC for NRA and help researchers optimize the conditions and generate highly selective electrocatalysts. In situ characterization techniques and operando experimental conditions must be facilitated so that the condition of the SAC can be determined after every experimental run. Owing to the highly reactive nature of TNMSACs, the operando characterization technique would provide a clearer picture of the catalytic states when incorporated into the catalytic research.

*Electrolyte Systems*. Elaborate studies should be conducted on the nature of the electrolyte systems. This would provide additional information and aid in understanding the NRA catalysis better. The effect of electrolyte pH, *i.e*., its acidic, basic or neutral nature, should be evaluated and its interaction with the TNMSAC should be given importance. The catalytic activity of TNMSACs in acidic, basic, and neutral buffer yields different results as a consequence of the variable interaction of the catalyst with the electrolyte. The difference in catalytic activity would also provide additional information on its NRA mechanism. Such studies must also be incorporated as a standard protocol to shine more light on the catalytic activity. Additional examinations should study the leaching of the catalyst and its stability in different systems.

*Stability and Reusability Issues*. Most of the literature in the field does not consider the critical aspect of long‐term stability tests. The reported experimental data lack long‐term stability evaluations like the state of the catalyst after 60, 90, or 180 days. The fate of the catalyst is important for incorporating these catalytic systems in industrial units. Hence, a standard protocol is missing for such experiments. A standardized experimental system should be developed to test the stability of the catalyst and then incorporated by the research community. Reusability of the catalyst is also not given much attention, which is at odds with the concept of sustainable research. Most of the works report that the catalyst was active till some cycles but do not provide an insight into what exactly happens to its composition over consecutive cycles. Similarly, characterization of the catalyst after completion of the catalytic cycles is also missing. A detailed characterization and analysis of the catalyst after each cycle would provide meaningful and stout knowledge on the changes and transitions of the phases of the catalyst.

*Scalability and Energy Efficiency*. To employ these reactions on a large scale, energy efficiency is another factor that requires deep consideration from the NRA catalysis point of view. In most literature, it is stated that single atoms are quite efficient in displaying high energy efficiency. However, they lack in explaining why single atoms show higher efficiency. They also fail to produce the proper mechanism of catalytic efficiency or produce improper information about the real catalytically active centers in the case of dual single atoms or diatoms/cocatalysts etc. Hence, detailed studies on the catalytic components to justify the energy efficiency of ammonia production will strengthen the overall research approach in the field of NRA catalysis. The large‐scale production of SAC is not given much attention. Most of the reported synthetic procedures are intricate, time‐consuming, and cannot be scaled up. More attention must be given to the direction of reaction scale‐up. Such gaps hinder the transfer of knowledge from the lab to industrial application. Because the electrochemical production of ammonia is an alternative to HBP, not being able to produce the catalyst at an industrial scale is a failure in the overall goal of NRA‐based research.

*Substrate Selection and Manufacturing*. (i) The idea of using bio‐based substrates for the fabrication of SACs has not been explored. Utilizing bioenzymes as substrates or matrices for the stabilization of SACs and for NRA is novel. This will not only expand knowledge about biocatalysts but also provide additional information for designing SACs. The introduction of other 2D materials like mxenes, germanene, borophene, arsenene, silicene, stanene, and phosphorene as base matrix material for the stabilization of the transition metal single atoms needs serious attention. This field of research has not been explored in detail. The variety of 2D materials and their interaction with single atoms would be an interesting topic for evaluation. (ii) Additive manufacturing should be given more emphasis for generating 3D‐printed SACs as it allows for decentralized fabrication.^[^ ^69^, ^78^ ^]^ 3D printing can be explored more extensively. Printing techniques like fused deposition modeling can be used for fabricating customized filaments, similarly, stereo lithographically assisted techniques can be used to fabricate catalyst surfaces with high resolution in micrometer range. The surface can be altered and the overall surface area can be enhanced using models with high resolution. These catalysts would deliver better NTA activity than conventionally printed catalysts. (iii) Laser patterning technology is a novel technology generally utilized to fabricate stand‐alone catalysts. This method opens up a wide scope for fabricating catalysts. Many substrates can be utilized for the fabrication of the catalyst. A simple process can be employed wherein the precursor transition metal salt solutions can be coated on the substrate surfaces followed by laser irradiation of different powers. After laser irradiation, there is a possibility the transition metal becomes pyrolyzed on the surface of the matrix to form TNMSAC.

*Toxicity Issues*. The concept of environmental impact assessment (EIA) has also not been taken into account while accessing the NRA conversion. What happens to the catalyst when it accidentally mixes with the freshwater bodies or is just discarded in the soil environment? How do they affect life forms when released into biosystems/ecosystems? Assessment of the catalyst has not been given prior importance nor it is included as part of the research. To address this gap, environmental impact assessment studies should be included after the NRA conversion experiments are concluded to follow the sustainable research model. As responsible scientists, we should try to incorporate such additional experiments along with lab experiments. Similarly, no safety data are published regarding TNMSACs. For instance, accidental human ingestion of the catalyst is the subject of an elaborate study. Hence, a general study should be conducted about their safety and hazardous effects on human beings. Based on these studies, precautions should be mentioned in order to minimize the risk of TNMSAC contamination.

To recapitulate, myriad attributes influence the overall NRA activity of TNMSACs: The nature of the central transition metal, type and heterogeneity of the stabilizing ligands, property of base matrix, polarity around the transition metal, d orbital electronic distribution, the effect of the adjacent TNMSAC (ensemble effect), the effect of stability (phase or structure changes), and the effect of intermetallic single/dual atom alloy formation. The initial step of nitrate adsorption on the catalyst surface is of paramount importance as it determines the outcome of NRA catalysis to a large extent. This factor has been corroborated by DFT simulation studies. Thus, this review shall serve as an insight into the strategic design of highly efficient and selective transition metal single atoms for conducting NRA catalysis while considering the salient aforementioned parameters that affect its activity.

## Conflict of Interest

There is no conflict of interest to declare.

## Author Contributions

S.S. conceptualized and designed the review article and executed the manuscript writing. M.P. originated the idea and supervised the writing work.
